# Adverse childhood experiences impact unemployment and inability to work among adults in the United States

**DOI:** 10.3389/fpsyg.2026.1630105

**Published:** 2026-02-26

**Authors:** Ray M. Merrill, Elaine N. Scott, Logan Sumsion

**Affiliations:** Department of Public Health, College of Life Sciences, Brigham Young University, Provo, UT, United States

**Keywords:** adverse childhood experiences, BRFSS, chronic illness, employment status, socioeconomic outcomes, workforce participation

## Abstract

**Introduction:**

In the context of employment, adults who had Adverse Childhood Experiences (ACE) may face unique barriers to employment due to physical, behavioral, and psychological effects. This study examines the relationship between ACEs and employment status, exploring how health behaviors and chronic illness may affect this association.

**Methods:**

This study draws on 2020–2024 Behavioral Risk Factor Surveillance System (BRFSS) data from 193,742 U. S. adults across 27 states, the District of Columbia, and 2 territories. Analyses involved bivariate analyses and regression techniques, adjusting for selected covariates.

**Results:**

Each of the 11 types of ACEs were significantly associated with lower odds of employment in adulthood, after adjusting for the demographic covariates. Even one ACE was sufficient to lower the odds of employment. As the number of ACEs increased, the odds of employment decreased. Children experiencing forced sexual contact had the lowest employment as adults. Among the specific covariates considered, education, household income, smoking, and chronic illness had the strongest effects on the association between the number of ACEs and employment.

**Discussion:**

These findings support existing evidence that ACEs contribute to long-term social and economic disadvantages. The study highlights the need for interventions that address the association between ACEs and unemployment.

## 1 Introduction

Adverse Childhood Experiences (ACEs) cause major disparities resulting in greater risk of mental illnesses (stress, anxiety, depression, suicide), chronic illnesses (obesity, cardiovascular disease, diabetes, cancer, cognitive disability), lower physical activity, substance abuse, problematic alcohol use, sexual risk taking, lower education and income, involvement in the justice system, and unemployment (Romm and Berg, 2024; Sebalo et al., 2023; Mahmood et al., 2023; Ward et al., 2022; Petersen et al., 2022; Kobrinsky and Siedlecki, 2022; Inoue et al., 2022; Kysar-Moon, 2021; Zhang and Monnat, 2021; Leza et al., 2021; Wiss and Brewerton, 2020; Dal Santo et al., 2020; Hughes et al., 2017; Merrick et al., 2017; Metzler et al., 2017; Monnat and Chandler, 2015; Sansone et al., 2012; Pilowsky et al., 2009; Felitti et al., 1998). In addition, ACEs are more common in communities with poverty, housing instability, neighborhood violence, and unemployment, thereby perpetuating a circular cycle adversely affecting future generations (Judd et al., 2023; Kysar-Moon, 2021; Zhang and Monnat, 2021; Bruner, 2017; Felitti et al., 1998). For example, higher rates of unemployment among adults with a history of ACEs are linked to adverse succession of disadvantages, with pathways involving lower education, mental health issues, incarceration, harmed family cohesion, and poorer health (Ratcliff et al., 2025; Jang and Kim, 2025; Erus et al., 2024; Metzler et al., 2017; Liu et al., 2012).

Disparities associated with ACEs stem from several interconnected theoretical frameworks, which include social determinants of health, neurobiology, and psychological processes (Camacho and Henderson, 2022; McLaughlin and Lambert, 2017; Metzler et al., 2017). These theories explain how ACEs can lead to a cascade of negative outcomes over the life course. Social and structural theories emphasize how the environment and systemic factors play a role in ACEs and resulting disparities. For example, some environments reflecting systematic inequality (poverty, discrimination, and community violence) compound the negative effects of ACEs (Haahr-Pedersen et al., 2020). In addition, life course theory suggests that early-life experiences accumulate over time, shaping adult health outcomes (Nurius et al., 2015). Biological and psychological mechanism theories focus on internal mechanisms in which ACEs affect individuals. For example, early adversities can begin psychological and physiological processes that negatively affect long-term health, whereas positive experiences can foster resilience (Haczkewicz et al., 2025). Childhood trauma can also disrupt brain development, affecting areas of the brain responsible for emotional management, self-regulation, cognitive function, and impulse control, causing poor stress management later in life (Nusslock and Miller, 2016).

Studies involving Behavior, Risk Factor, Surveillance System (BRFSS) data have found a strong association between people with more ACEs and higher levels of unemployment or inability to work (Swedo et al., 2023; Giano et al., 2020; Metzler et al., 2017). Lifelong economic burden due to lost earnings is much higher in adults with multiple ACEs, which negatively affects health and opportunity (Merrick et al., 2018; Metzler et al., 2017; Font and Maguire-Jack, 2015). The connection between ACEs and unemployment is often mediated through lower education and income, marital status, and social support (Liu et al., 2012).

Studies involving other data sources have also assessed ACEs and employment, along with potential mediating factors. A study involving National Longitudinal Study of Adolescent to Adult Health data found educational attainment and depressive symptoms, followed by incarceration, were the major prominent mediators between ACEs and unemployment (Jang and Kim, 2025). In a study involving Black males in the Midwest region of the U. S., ACEs negatively affected ability to attain employment and drug abuse and depressive symptoms mediated this relationship (Topitzes et al., 2016). In a study conducted in northeastern Japan, a significant association was found between individuals undergoing medical treatment for chronic disease (stroke, cancer, myocardial infarction and angina) and unemployment, but only among those with a high level of psychological distress and/or poorer levels of daily life activity (Nakaya et al., 2016).

Using data from the 2020–2024 BRFSS, the current study further examined the association between ACEs and adult employment status. The results are more current, comprehensive, and extend previous research by assessing associations between specific ACE items and employment status and evaluating the effect of certain covariates on the association. The association between the number of ACEs and employment status was also assessed, along with the effect of certain covariates on the relationship.

## 2 Materials and methods

### 2.1 Data

This study is based on data from the 2020–2024 Behavior Risk Factor Surveillance System (BRFSS). The BRFSS is a nationwide random probability telephone survey conducted annually that collects individual-level data from U. S. states, territories, and the District of Columbia on health behaviors, chronic health conditions, and preventive service usage. It uses a cross-sectional design with standardized questionnaires and random probability samples of the adult population (ages 18 and older). Three parts make up the BRFSS survey: (1) standard core questions; (2) rotating core questions; and (3) optional modules (Centers for Disease Control, 2013). Median response rates for all participating areas were 45% in 2020, 44% in 2021, 45% in 2022, 47% in 2023, and 46% in 2024 (Centers for Disease Control, 2021, 2022, 2023, 2024, 2025a). All participants provided informed verbal consent prior to the interview. Details of the BRFSS survey design, questionnaires, and data collection method are available elsewhere (Centers for Disease Control, 2025b).

Data analyses included 27 states (Alabama, Arizona, Arkansas, Delaware, Florida, Georgia, Hawaii, Idaho, Iowa, Kentucky, Mississippi, Missouri, Montana, Nevada, New Hampshire, New Jersey, North Dakota, Oregon, Rhode Island, South Carolina, South Dakota, Tennessee, Texas, Utah, Virginia, Wisconsin, Wyoming) and the District of Columbia, and two U. S. territories (Puerto Rico, Virgin Islands), which included questions about Adverse Childhood Experiences. The total number of participants in these areas was 331,758. Analyses included 193,742, of which 158,131 were currently employed, 13,942 were unemployed, and 21,669 were unable to work. Those not included in this study were homemakers (*n* = 13,614), students (*n* = 7,239), retired individuals (*n* = 114,413), and those who refused to indicate their work status (*n* = 2,750).

### 2.2 Measures

Demographic variables included age, sex, race/ethnicity, marital stat us, education, and household income (see Table 1). Health-risk behavior, body mass index (BMI) weight classifications, and chronic illness variables were also considered (see Table 2). The health-risk behavior variables were exercise, smoking status, heavy drinking, binge drinking, and cannabis use. Exercise in the past 30 days was based on the question: “During the past month, other than your regular job, did you participate in any physical activities or exercises such as running, calisthenics, golf, gardening, or walking for exercise?” Being a current smoker was based on whether they were an everyday smoker, occasional smoker, former smoker, or non-smoker. Heavy drinkers refer to adult men having more than 14 drinks per week and adult women having more than 7 drinks per week. Binge drinkers refer to males having five or more drinks on one occasion and females having four or more drinks on one occasion.

**Table 1:** Employment status by demographic variables in the United States.

			Employed	Unemployed	Unable to work	Rao-Scott Chi-Square	Odds ratio
	No.	Col % (SE)^*^	Row % (SE)^*^	Row % (SE)^*^	Row % (SE)^*^	Pr > |t|	(95% CI)^*†^
Age							
18–24	12,552	10.6 (0.2)	82.1 (0.7)	14.7 (0.7)	3.2 (0.3)	<0.0001	1.00
25–34	28,021	19.8 (0.2)	85.9 (0.4)	9.8 (0.4)	4.3 (0.2)		0.96 (0.84–1.09)
35–44	36,396	21.1 (0.2)	86.3 (0.4)	7.8 (0.3)	5.9 (0.3)		0.81 (0.71–0.93)
45–54	41,926	20.8 (0.2)	83.3 (0.4)	6.7 (0.3)	10.0 (0.3)		0.59 (0.52–0.68)
55–64	47,789	19.5 (0.2)	73.9 (0.4)	6.9 (0.2)	19.2 (0.4)		0.36 (0.32–0.41)
≥65	27,058	8.2 (0.1)	68.5 (0.7)	6.2 (0.4)	25.3 (0.7)		0.34 (0.30–0.39)
Sex							
Men	97,001	52.2 (0.2)	83.8 (0.3)	7.8 (0.2)	8.4 (0.2)	<0.0001	1.00
Women	96,741	47.8 (0.2)	78.6 (0.3)	9.0 (0.2)	12.4 (0.2)		0.76 (0.72–0.80)
Race/ethnicity							
NH White	134,469	57.0 (0.2)	83.9 (0.2)	6.6 (0.1)	9.5 (0.1)	<0.0001	1.00
NH Black	19,532	15.4 (0.2)	74.4 (0.6)	11.1 (0.4)	14.6 (0.4)		0.76 (0.71–0.83)
NH other	16,979	8.0 (0.1)	81.4 (0.7)	9.8 (0.5)	8.8 (0.5)		0.78 (0.71–0.86)
Hispanic	19,149	17.8 (0.2)	79.2 (0.6)	11.1 (0.5)	9.7 (0.5)		1.18 (1.07–1.30)
Unknown	3,613	1.8 (0.1)	78.9 (1.5)	8.9 (1.3)	12.2 (1.0)		0.96 (0.77–1.19)
Marital status							
Married/Cohab	109,839	55.6 (0.2)	86.9 (0.2)	6.0 (0.2)	7.1 (0.2)	<0.0001	1.00
Previously M	41,513	18.2 (0.2)	68.4 (0.5)	9.3 (0.3)	22.3 (0.4)		0.68 (0.63–0.73)
Never M	41,045	25.6 (0.2)	78.3 (0.4)	12.8 (0.4)	8.9 (0.3)		0.67 (0.62–0.73)
Unknown	1,345	0.7 (0.0)	80.7 (2.2)	10.1 (1.9)	9.2 (1.2)		1.06 (0.77–1.47)
Education							
<HS	12,422	11.1 (0.2)	58.5 (0.9)	13.4 (0.6)	28.1 (0.7)	<0.0001	1.00
HS	49,693	27.7 (0.2)	77.1 (0.4)	10.5 (0.3)	12.4 (0.3)		1.86 (1.70–2.03)
Some college	53,619	30.5 (0.2)	82.3 (0.4)	8.2 (0.3)	9.4 (0.2)		2.22 (2.01–2.44)
College	77,487	30.3 (0.2)	92.6 (0.2)	4.7 (0.2)	2.7 (0.1)		4.04 (3.66–4.46)
Unknown	521	0.3 (0.0)	71.8 (5.2)	9.5 (2.7)	18.7 (5.1)		1.47 (0.74–2.93)
Household income							
<50 K	65,829	34.7 (0.2)	65.9 (0.4)	14.1 (0.3)	20.0 (0.3)	<0.0001	1.00
50 K–<100 K	68,673	32.6 (0.2)	93.0 (0.2)	4.3 (0.2)	2.7 (0.1)		4.87 (4.47–5.32)
100 K–<200 K	24,031	12.9 (0.1)	96.7 (0.2)	1.9 (0.2)	1.4 (0.2)		9.62 (8.25–11.22)
≥200 K	7,999	4.8 (0.1)	97.4 (0.4)	1.5 (0.2)	1.0 (0.3)		11.17 (8.20–15.22)
Unknown	27,210	14.9 (0.2)	73.0 (0.6)	11.9 (0.4)	15.1 (0.4)		1.23 (1.15–1.32)
Year							
2000	78,580	36.6 (0.1)	79.9 (0.3)	9.7 (0.3)	10.5 (0.2)	<0.0001	1.00
2001	33,661	14.2 (0.1)	82.6 (0.3)	7.4 (0.2)	10.0 (0.2)		1.07 (1.00–1.14)
2002	28,271	12.8 (0.1)	82.2 (0.6)	7.0 (0.4)	10.8 (0.4)		0.99 (0.90–1.09)
2003	31,843	21.8 (0.2)	82.0 (0.4)	7.8 (0.3)	10.2 (0.3)		0.92 (0.85–0.99)
2004	21,387	14.6 (0.2)	81.8 (0.5)	8.3 (0.4)	10.0 (0.4)		0.90 (0.82–0.99)
Total	193,742	100.0 (0.0)	81.3 (0.2)	8.4 (0.1)	10.3 (0.1)		

Data source: BRFSS 2020–2024. SE, Standard Error; CI, Confidence Interval.

^*^Weighted estimates, based on the complex sampling design used.

^†^Adjusted for the variables in the table.

**Table 2:** Employment status by selected behavior variables and chronic health conditions.

			Employed	Unemployed	Unable to work	Rao-Scott	Odds ratio
	No.	% (SE)^*^	% (SE)^*^	% (SE)^*^	% (SE)^*^	Pr > |t|	(95% CI)^*†^
Exercise in past 30 days							
No	46,598	24.4 (0.2)	67.6 (0.5)	9.7 (0.3)	22.7 (0.4)	<0.0001	1.00
Yes	146,828	75.4 (0.2)	85.8 (0.2)	7.9 (0.2)	6.3 (0.1)		1.95 (1.78–2.14)
Unknown	316	0.2 (0.0)	58.0 (7.0)	16.8 (4.8)	25.2 (5.7)		1.87 (0.94–3.74)
Smoking status							
Every day	21,688	10.9 (0.1)	68.5 (0.6)	12.2 (0.4)	19.4 (0.5)	<0.0001	0.62 (0.54–0.71)
Occasional	8,430	4.9 (0.1)	71.7 (1.0)	11.5 (0.7)	16.8 (0.8)		0.59 (0.50–0.70)
Former	46,034	22.2 (0.2)	80.0 (0.4)	7.5 (0.3)	12.5 (0.3)		0.79 (0.71–0.87)
Never	116,447	61.4 (0.2)	84.8 (0.2)	7.8 (0.2)	7.4 (0.2)		1.00
Unknown	1,143	0.6 (0.0)	76.0 (2.3)	9.3 (1.8)	14.7 (1.7)		0.88 (0.53–1.45)
Heavy drinker							
No	176,180	90.7 (0.1)	81.0 (0.2)	8.3 (0.1)	10.7 (0.1)		1.00
Yes	13,674	7.0 (0.1)	85.5 (0.6)	8.4 (0.5)	6.1 (0.4)		1.32 (1.11–1.56)
Unknown	3,888	2.3 (0.1)	80.1 (1.3)	10.0 (1.0)	9.8 (0.9)		1.39 (1.05–1.84)
Binge drinker							
No	156,973	80.2 (0.2)	79.9 (0.2)	8.5 (0.2)	11.6 (0.2)	<0.0001	1.00
Yes	32,907	17.5 (0.2)	87.9 (0.4)	7.7 (0.3)	4.5 (0.2)		1.55 (1.36–1.75)
Unknown	3,862	2.3 (0.1)	78.1 (1.4)	10.8 (1.1)	11.1 (1.0)		1.43 (1.11–1.83)
Monthly cannabis use							
No	55,372	82.6 (0.3)	83.5 (0.3)	7.4 (0.2)	9.1 (0.2)	0.0010	1.00
Yes	8,574	16.1 (0.3)	77.4 (0.8)	11.8 (0.6)	10.8 (0.5)		0.73 (0.65–0.81)
Unknown	664	1.3 (0.1)	83.3 (2.2)	6.4 (1.3)	10.3 (1.9)		1.28 (0.90–1.80)
Body mass index							
Underweight	2,717	1.5 (0.1)	69.3 (1.7)	13.0 (1.4)	17.8 (1.3)	<0.0001	0.74 (0.56–0.97)
Normal weight	48,929	25.9 (0.2)	82.9 (0.4)	8.9 (0.3)	8.2 (0.2)		1.00
Overweight	63,050	32.0 (0.2)	84.2 (0.3)	7.5 (0.2)	8.3 (0.2)		1.08 (0.96–1.22)
Obese	67,317	34.2 (0.2)	78.4 (0.3)	8.1 (0.2)	13.5 (0.3)		0.84 (0.76–0.94)
Unknown	11,729	6.4 (0.1)	78.2 (0.9)	11.2 (0.7)	10.6 (0.7)		1.09 (0.91–1.31)
Chronic illness							
0	88,316	49.3 (0.2)	84.4 (0.4)	6.7 (0.3)	8.9 (0.3)	<0.0001	1.00
1–2	82,003	40.5 (0.2)	82.8 (0.3)	7.9 (0.2)	9.2 (0.2)		0.49 (0.45–0.52)
3–4	18,775	8.2 (0.1)	79.8 (0.5)	9.4 (0.4)	10.8 (0.3)		0.18 (0.15–0.20)
≥5	4,648	2.0 (0.1)	74.4 (0.5)	11.2 (0.4)	14.4 (0.4)		0.06 (0.05–0.08)

Data source: BRFSS 2020–2024. SE, Standard Error; CI, Confidence Interval.

^*^Weighted estimates, based on the complex sampling design used.

^†^Separate models were run for each variable, adjusting for age, sex, race/ethnicity, marital status, education, annual household income, and year.

Exercise in the past 30 days is based on the question: “During the past month, other than your regular job, did you participate in any physical activities or exercises such as running, calisthenics, golf, gardening, or walking for exercise?” Heavy drinkers refer to adult men having more than 14 drinks per week and adult women having more than 7 drinks per week. Binge drinkers refer to males having five or more drinks on one occasion and females having four or more drinks on one occasion.

Not all the 30 U. S. areas in which the BRFSS surveys were conducted included questions about ACEs during 2020–2024 asked about cannabis use. However, 15 (Delaware, Hawaii, Idaho, Kentucky, Mississippi, Nevada, New Hampshire, North Dakota, Oregon, Rhode Island, South Carolina, Utah, Virginia, Wyoming, Virgin Islands) areas did. The specific question asked was: “During the past 30 days, on how many days did you use marijuana or cannabis?” There were 64,610 respondents. Respondents who used cannabis at least once in the past 30 days were defined as monthly users. Of 8,574 who used cannabis at least once in the past 30 days, 10% used it once and 41% used it daily. Mean and median number of days of use were 17 and 20, respectively.

The four BMI categories were underweight (BMI < 18.50), normal weight (18.5 ≤ BMI < 25.00), overweight (25.00 ≤ BMI < 30.00), and obese (30.00 ≤ BMI < 99.99).

Ten types of chronic diseases were considered. Participants were asked if they were ever told or had a heart attack (myocardial infarction); angina or coronary heart disease; stroke; asthma; melanoma or any type of cancer (not including skin cancer); chronic obstructive pulmonary disease, emphysema, or chronic bronchitis; depressive disorder; kidney disease; arthritis, rheumatoid arthritis, gout, lupus, or fibromyalgia; or diabetes. A variable was created representing the number of these diseases: 0, 1–2, 3–4, ≥5. The level of unknown/missing information on these chronic illnesses ranged from 0.24 to 0.64%. These cases were combined with 0.

Eleven ACE questions and answer options were considered (see Table 3). These ACE items are routinely included in the BRFSS. The items cover a range of topics, including emotional abuse, physical abuse, and sexual abuse.

**Table 3:** Odds of employment by types of adverse child hood experience (ACE) in the United States.

			Employed	Unemployed	Unable to work	Rao-Scott	Odds ratio
	No.	% (SE)^*^	% (SE)^*^	% (SE)^*^	% (SE)^*^	Pr > |t|	(95% CI)^*†^
Did you live with anyone who was depressed, mentally ill, or suicidal?							
No	148,731	76.2 (0.2)	82.2 (0.2)	7.9 (0.2)	9.9 (0.2)	<0.0001	1.00
Yes	40,702	21.6 (0.2)	78.7 (0.4)	9.9 (0.3)	11.4 (0.3)		0.67 (0.63–0.71)
Unknown	4,309	2.2 (0.1)	75.5 (1.3)	9.9 (1.2)	14.6 (0.9)		0.76 (0.64–0.90)
Did you live with anyone who was a problem drinker or alcoholic?							
No	141,250	73.0 (0.2)	82.7 (0.2)	8.0 (0.2)	9.3 (0.2)	<0.0001	1.00
Yes	49,119	25.2 (0.2)	77.5 (0.4)	9.5 (0.3)	13.0 (0.3)		0.77 (0.73–0.82)
Unknown	3,373	1.7 (0.1)	77.4 (1.6)	9.7 (1.4)	12.9 (1.0)		0.87 (0.71–1.08)
Did you live with anyone who used illegal street drugs or who abused prescription medications?							
No	164,839	83.3 (0.2)	82.0 (0.2)	8.0 (0.2)	10.0 (0.2)	<0.0001	1.00
Yes	25,181	14.7 (0.2)	78.2 (0.5)	10.4 (0.4)	11.4 (0.4)		0.78 (0.72–0.83)
Unknown	3,722	2.0 (0.1)	74.8 (1.6)	11.2 (1.5)	14.0 (0.9)		0.74 (0.62–0.89)
Did you live with anyone who served time or was sentenced to serving time in a prison, jail, or other correctional facility?							
No	171,918	86.5 (0.2)	82.2 (0.2)	7.8 (0.1)	10.0 (0.1)	<0.0001	1.00
Yes	18,437	11.7 (0.2)	75.4 (0.6)	12.2 (0.5)	12.4 (0.5)		0.75 (0.69–0.82)
Unknown	3,387	1.8 (0.1)	77.0 (1.7)	10.4 (1.6)	12.6 (1.0)		0.83 (0.67–1.02)
Were your parents separated or divorced?							
No	125,958	59.9 (0.2)	83.3 (0.2)	7.1 (0.2)	9.5 (0.2)	<0.0001	1.00
Yes	60,124	35.3 (0.2)	78.9 (0.3)	9.9 (0.2)	11.3 (0.2)		0.82 (0.77–0.87)
Parents not married	4,219	2.4 (0.1)	74.1 (1.4)	13.4 (1.2)	12.5 (0.9)		0.75 (0.61–0.91)
Unknown	3,441	2.5 (0.1)	73.3 (1.6)	12.8 (1.5)	13.9 (1.0)		0.77 (0.64–0.92)
How often your parents or adults in your home ever slap, kick, punch or beat each other up?							
Never	152,523	77.3 (0.2)	82.9 (0.2)	7.8 (0.2)	9.3 (0.2)	<0.0001	1.00
Once	8,185	4.6 (0.1)	78.3 (1.0)	11.0 (0.8)	10.6 (0.7)		0.79 (0.70–0.90)
More than once	26,316	14.4 (0.2)	75.3 (0.5)	10.4 (0.4)	14.4 (0.4)		0.71 (0.67–0.77)
Unknown	6,718	3.7 (0.1)	73.8 (1.2)	10.1 (1.0)	16.1 (0.9)		0.77 (0.67–0.88)
Not including spanking (before age 18), how often did a parent or adult in your home ever hit, beat, kick, or physically hurt you in any way? Was it –							
Never	139,172	70.0 (0.2)	83.1 (0.2)	7.6 (0.2)	9.2 (0.2)	<0.0001	1.00
Once	11,963	6.3 (0.1)	80.7 (0.7)	9.4 (0.6)	9.9 (0.5)		0.88 (0.79–0.98)
More than once	36,991	20.6 (0.2)	76.0 (0.4)	10.3 (0.3)	13.7 (0.3)		0.70 (0.66–0.75)
Unknown	5,616	3.1 (0.1)	76.1 (1.3)	10.5 (1.0)	13.4 (0.9)		0.79 (0.68–0.92)
How often did a parent or adult in your home ever swear at you, insult you, or put you down?							
Never	115,606	58.4 (0.2)	82.3 (0.2)	7.6 (0.2)	10.0 (0.2)	<0.0001	1.00
Once	10,589	5.5 (0.1)	83.0 (0.8)	9.1 (0.7)	7.9 (0.5)		0.91 (0.80–1.04)
More than once	61,186	32.8 (0.2)	79.5 (0.3)	9.5 (0.3)	11.0 (0.2)		0.71 (0.67–0.75)
Unknown	6,361	3.3 (0.1)	77.7 (1.1)	9.5 (1.0)	12.8 (0.8)		0.82 (0.71–0.95)
How often did anyone, at least 5 years older than you or an adult, ever touch you sexually?							
Never	162,896	83.6 (0.2)	82.8 (0.2)	7.9 (0.2)	9.2 (0.1)	<0.0001	1.00
Once	7,989	4.3 (0.1)	76.8 (0.9)	10.0 (0.7)	13.2 (0.7)		0.77 (0.68–0.87)
More than once	16,505	8.6 (0.1)	71.2 (0.7)	10.9 (0.5)	17.9 (0.6)		0.59 (0.54–0.65)
Unknown	6,352	3.5 (0.1)	74.9 (1.2)	10.8 (1.0)	14.3 (0.8)		0.77 (0.67–0.88)
How often did anyone, at least 5 years older or an adult, try to make you touch them sexually?							
Never	168,480	86.2 (0.2)	82.5 (0.2)	8.0 (0.2)	9.5 (0.1)	<0.0001	1.00
Once	6,349	3.5 (0.1)	78.3 (1.0)	10.1 (0.7)	11.6 (0.8)		0.91 (0.79–1.05)
More than once	12,475	6.7 (0.1)	70.7 (0.8)	11.1 (0.6)	18.2 (0.6)		0.58 (0.53–0.63)
Unknown	6,438	3.6 (0.1)	74.4 (1.2)	10.6 (1.0)	15.0 (0.8)		0.78 (0.67–0.90)
How often did anyone at least 5 years older than you or an adult, force you to have sex?							
Never	175,970	90.3 (0.1)	82.6 (0.2)	8.0 (0.1)	9.4 (0.1)	<0.0001	1.00
Once	3,748	2.2 (0.1)	71.0 (1.5)	12.4 (1.1)	16.7 (1.1)		0.67 (0.56–0.79)
More than once	7,524	4.0 (0.1)	63.8 (1.1)	12.6 (0.8)	23.6 (0.9)		0.50 (0.45–0.56)
Unknown	6,500	3.6 (0.1)	74.9 (1.2)	10.3 (1.0)	14.9 (0.8)		0.80 (0.69–0.92)

BRFSS 2020–2024. SE, Standard Error; CI, Confidence Interval.

^*^Weighted estimates, based on the complex sampling design used.

^†^Separate models were run for each variable, adjusting for age, sex, race/ethnicity, marital status, education, annual household income, and year.

### 2.3 Statistical analysis

The data was described using numbers, relative frequencies, and standard errors. Estimates were determined by considering the survey stratum, primary sampling units, and sampling weights. We used logistic regression to estimate the odds of being employed according to selected demographics, health-risk behaviors, body weight classification, and chronic illnesses. Logistic regression was also used to estimate the odds of being employed according to the ACE items and the number of ACEs. The odds ratios estimated in these models were adjusted for age, sex, race/ethnicity, marital status, education, annual household income, and year. Regression was used to estimate the mean number of ACEs according to demographics, health-risk behaviors, body weight classification, and chronic illnesses. Statistical independence between categorical variables was assessed for significance using the Rao-Scott Chi-Square Test. Confidence intervals were presented with the odds ratio, indicating statistical significance when they did not overlap 1. Statistical significance was based on two-sided tests at the 0.05 level. Statistical analyses were conducted using Statistical Analysis System (SAS) software, version 9.4 (SAS Institute Inc., Cary, NC, United States, 2016).

## 3 Results

The distributions of the demographic variables appear in Table 1. About 81% were employed, 9% unemployed, and 10% unable to work. The relationship between demographic variables and employment status is also presented. In the adjusted model, the odds of being employed decreased with age, was higher for men, non-Hispanic Whites and Hispanics, married (or cohabitating), and in those with higher education and income.

**Figure 1:**
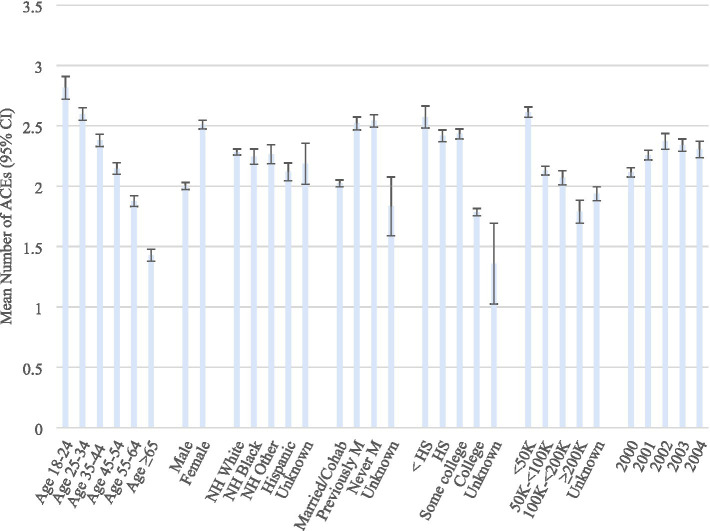
Mean number of ACEs according to demographics. BRFSS 2020–2024. ACEs, Adverse Childhood Experiences; CI, Confidence interval. Weighted odds ratios, based on the complex sampling design used.

The mean number of different types of ACEs was 2.2 (SE = 0.01). These are presented across the levels of the demographic variables in Figure 1. The mean numbers of different types of ACEs were significantly greater in younger age groups, women, non-Hispanics, previously or never married, and those with lower education and income.

Employment status by selected health-risk behaviors, body weight classifications, and chronic illnesses appears in Table 2. In the adjusted models, the odds of being employed were significantly lower for those who did not exercise in the past 30 days, smoked daily, occasionally, or formerly, used cannabis once or more in the past month, or had one or more chronic illnesses. The odds of heavy drinking or binge drinking were significantly higher among those who were employed.

**Figure 2:**
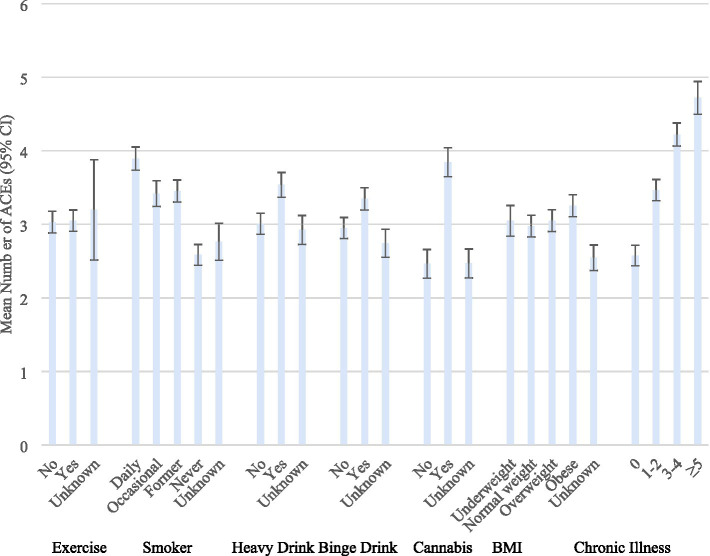
Mean number of ACEs according to health-risk behaviors, weight classifications, and chronic illnesses. BRFSS 2020–2024. ACEs, Adverse Childhood Experiences; CI, Confidence interval. Weighted odds ratios, based on the complex sampling design used.

The mean number of different types of ACEs are presented according to the levels of health-risk behaviors, body weight classifications, and chronic illnesses in Figure 2. The means were significantly greater among those with a history of smoking, heavy drinking, binge drinking cannabis use, obesity, and chronic illnesses. The significant associations between ACEs and these variables was most pronounced for chronic illnesses.

The prevalence of 11 ACEs is presented in Table 3. The most common ACEs involved parents who were separated, divorced, or an unmarried couple, and having a parent or adult in the home swear at you, insult you, or put you down. The next most common ACEs involved living with anyone who was depressed, mentally ill, or suicidal, a problem drinker or alcoholic, and being physically hurt by a parent or adult in your home. The least common ACEs involved having anyone, at least 5 years older, or an adult, ever touch you sexually, force you to touch them sexually, or force you to have sex. Experiencing any ACEs was significantly associated with lower odds of employment. Having a sexual relationship with someone at least 5 years older or an adult during childhood, especially more than once, had the strongest negative association with being employed.

**Figure 3:**
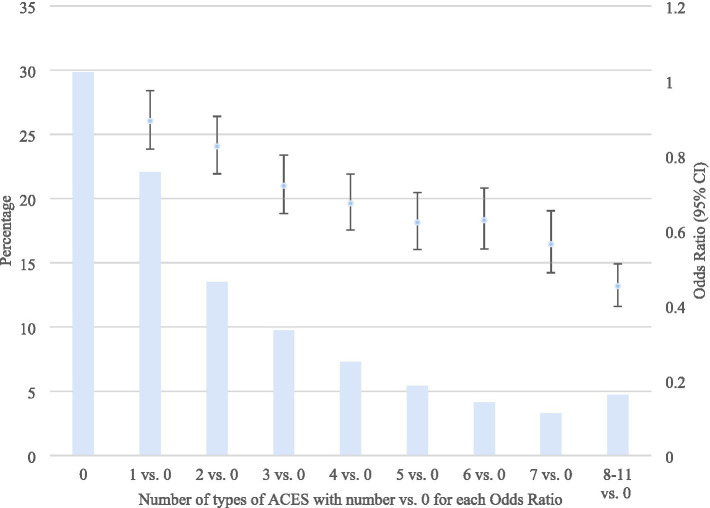
Odds of employment (vs. otherwise) according to the number of ACEs (vs. 0). BRFSS 2020–2024. ACEs, Adverse Childhood Experiences; CI, Confidence Interval. Weighted odds ratios, based on the complex sampling design used, adjusted for age, sex, race/ethnicity, marital status, education, annual household income, and year.

As the number of ACEs increased, the odds of employment consistently decreased, after adjusting for the demographic covariates (Figure 3). Even 1 ACE was sufficient to significantly lower the odds of employment. Those with 8–11 ACEs were less than half as likely to be employed as those with no ACEs.

**Figure 4:**
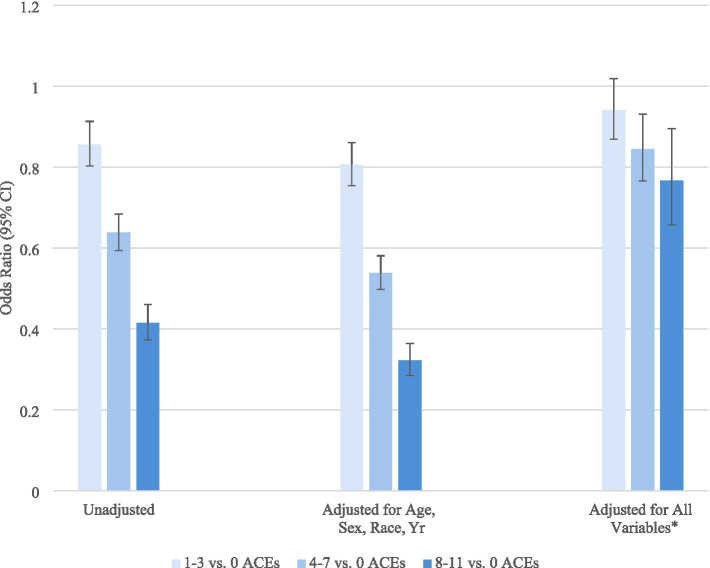
Odds of employment (vs. otherwise) according to the number of adverse childhood experience (ACE) (vs. 0) and adjusted covariates. BRFSS, 2020–2024. ACEs, Adverse Childhood Experiences; CI, Confidence Interval. *Weighted estimates, based on the complex sampling design used. All variables include age, sex, race/ethnicity, marital status, education, annual household income, year, exercise, smoking status, heavy drinking, binge drinking, monthly cannabis use, weight classifications, and chronic illnesses.

To further assess the effect of the covariates on the association between number of ACEs and employment, we compared the odds of employment for those with 1–3 vs. 0 ACEs, 4–7 vs. 0 ACEs, and 8–11 vs. 0 ACEs (Figure 4). The odds ratios were significantly lower after adjusting for age, sex, race/ethnicity, and year. Additional adjustment for health-risk behaviors, body weight classifications, and chronic illnesses explained some of associations, more so for those with a higher number of ACEs. However, for those experiencing ≥4 ACEs, the lower odds of employment could not be fully explained by the covariates included.

**Figure 5:**
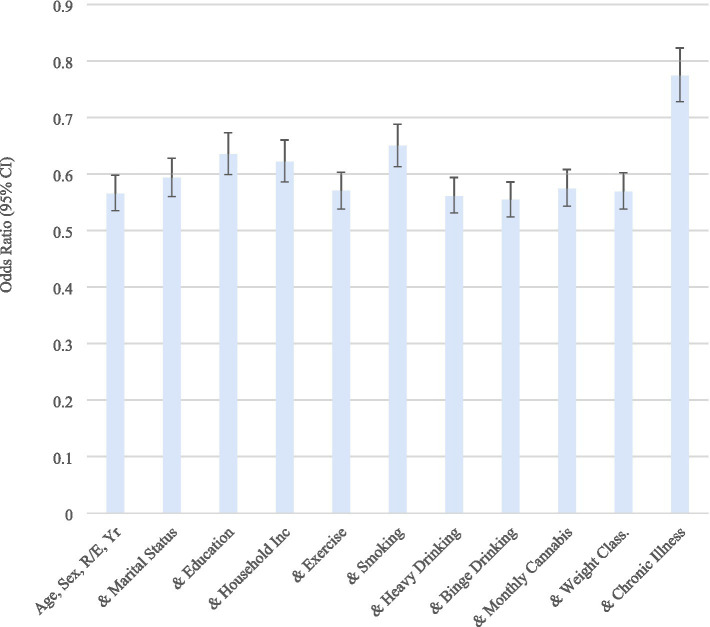
Odds of employment (vs. otherwise) according to ≥4 vs. 0–3 ACEs, adjusting for certain demographics, health-risk behaviors, body weight classifications, and chronic illnesses. BRFSS 2020–2024. ACEs, Adverse Childhood Experiences; CI, Confidence Interval. Weighted Odds Ratios, based on the complex sampling design used. Each model included age, sex, race/ethnicity, and year. For example, “& Smoking” means the model contained age, sex, race/ethnicity, year, and smoking.

Finally, we compared the odds of employment for those with ≥4 vs. 0–3 ACEs, adjusting for certain demographics, health-risk behaviors, body weight classifications, and chronic illnesses (Figure 5). Each of the models was significant. Education, household income, smoking status, and chronic illness significantly explained the lower odds of employment for those with greater number of ACEs beyond the effects of age, sex, race/ethnicity, and year. In other words, these variables appear to have a significant mediating effect.

## 4 Discussion

The current study used 2020–2024 BRFSS data to evaluate the association between specific ACE items and the number of ACEs with adult employment status. The effects of certain demographics, health-risk behaviors, body weight classifications, and chronic illnesses on these associations were also explored. Each demographic variable was significantly associated with both employment status and mean number of ACEs. In addition, each health-risk behavior, body weight classification, and chronic health condition variable was significantly associated with employment status and mean number of ACEs, except exercise. Hence, these variables were treated as covariates in the study.

The relationships between the demographic variables and employment status are consistent with other reports (U.S. Bureau of Labor Statistics, 2024, 2025; U.S. Department of Labor, Women's Bureau, 2024). The relationships between the demographic variables and mean number of ACEs are also consistent with the literature (Swedo et al., 2023; Ward et al., 2022). Some of these variables are confounders (age, sex, race/ethnicity, year), while others may be affected by ACEs (marital status, education, and household income) and, in turn, impact employment status.

The observation that poor health behaviors, obesity, and chronic conditions are more prevalent among individuals with disrupted employment has been observed previously (Jang and Kim, 2025; Ward, 2015). Physical inactivity, smoking, and cannabis use were all elevated among those who were unemployed or unable to work. Substance use has a well-documented relationship with employment (Nolte-Troha et al., 2023). The higher odds of employment among those who are heavy drinkers or binge drinkers is unclear. It may be that employed individuals are more likely to afford drinking. Social work environments or stressful jobs may also promote drinking. Further research to understand these findings is needed. Additionally, individuals with more chronic conditions were less likely employed, as consistent with their being unable to or less effective in work (Nakaya et al., 2016).

The current study found that higher mean number of ACEs were associated with current or former smoking, heavy alcohol drinking, binge drinking, and cannabis use, as consistent with previous research (Sebalo et al., 2023; Giano et al., 2020; Martinasek et al., 2021; Monnat and Chandler, 2015; Pilowsky et al., 2009). Individuals with a history of ACEs may be more likely to engage in these behaviors to cope with resulting stress, emotional distress, or trauma (Dal Santo et al., 2020; Inoue et al., 2022; Romm and Berg, 2024). Furthermore, childhood adversity can contribute to physiological and psychological changes that influence decision-making and risk perception, thereby reinforcing these health behaviors (Monnat and Chandler, 2015; Wang et al., 2019). Other research has found that early life adversity amplifies chronic low-grade inflammation, contributing to adiposity, insulin resistance, and other pre-disease conditions, along with self-medicating behaviors like smoking, drug use, and high-fat diets (Nusslock and Miller, 2016; Nurius et al., 2015).

Higher mean number of ACEs also occurred in underweight and obese individuals, as well as in those with more chronic health conditions. The link between ACEs and obesity is well established (Mahmood et al., 2023), with proposed mechanisms including social disruption, health behaviors, and chronic stress responses (Wiss and Brewerton, 2020). The link between ACEs and underweight in adulthood is not well established, but previous research has found ACEs to increase the risk of being underweight in children, with the likely mechanisms including eating disorders and malnutrition (Hanć et al., 2022). Finally, the positive link between ACEs and chronic health conditions is also well known (Erus et al., 2024; Hughes et al., 2017; Merrick et al., 2017; Metzler et al., 2017; Edwards et al., 2003).

Any of the 11 ACEs was associated with significantly lower employment in adulthood, and even one ACE was sufficient to significantly lower the odds of future employment. Consistently, another study found that those who had experienced any maltreatment during childhood were twice as likely to be unemployed (Sansone et al., 2012). Childhood sexual abuse had the greatest negative impact on later employment. Those who experienced forced sexual contact more than once showed the strongest negative association. Similarly, verbal and physical abuse, household substances use and living with someone who was incarcerated were all significantly associated with reduced odds of employment in adulthood. This shows that the absence of a safe and supportive adult during childhood was a strong predictor of employment in adulthood, underscoring the long-term impact of early relationships and environmental instability.

A clear dose–response relationship was observed between a higher number of ACEs and lower odds of employment in adulthood. Some of the associations between higher number of ACES and lower odds of employment were explained by education, household income, smoking, and chronic illness. Other research has similarly found the connection between ACEs and unemployment to be mediated through lower education and income (Ratcliff et al., 2025; Jang and Kim, 2025; Merrick et al., 2018; Liu et al., 2012). Further, research has found that drug abuse and chronic depression mediate the relationship between ACEs and unemployment (Jang and Kim, 2025; Topitzes et al., 2016).

While our results explained much about the association between ACEs and employment, it could not explain it all. Additional variables that may have further helped explain the association could include parental socioeconomic status (SES) and social support. Lower parental SES is associated with increased ACEs and the likelihood of later unemployment (Haahr-Pedersen et al., 2020). Social support has also been found to mediate the relationship between ACEs and unemployment (Jang and Kim, 2025; Liu et al., 2012).

### 4.1 Limitations

Adult participants in this study were asked to recall ACE events, which could have occurred many years earlier. The level of recall bias is unknown; however, greater accuracy of responses is assumed because the survey was anonymous. A systematic review of publications found that self-reported BRFSS data was both reliable and valid (Pierannunzi et al., 2013). External validity may be an issue because not all U. S. were asked questions about ACEs. Nevertheless, 30 U. S. areas were considered. It is assumed that the decision to ask ACE questions was not influenced by the level of ACEs in the areas. Another limitation is that we did not have information about parental SES and social support, which may have been helpful in further explaining the associations assessed in this study.

## 5 Conclusion

This study extends previous research by analyzing the relationship between specific ACEs and the number of ACEs with employment status using 2020–2024 Behavioral Risk Factor Surveillance System (BRFSS) data. The effects of specific covariates on the associations were examined. Each of the 11 types of ACEs significantly related to lower employment, after adjusting for the demographic covariates. Even one ACE was sufficient to lower the odds of employment as an adult. Children experiencing forced sexual contact had the lowest employment as adults. Living with a parent or adult who was verbally or physically abusive, used substances, or was incarcerated also was associated with lower employment in adulthood. Hence, assuring a safe and supportive environment for children is critical in promoting employment in adulthood. Further, younger aged adults and women had significantly higher mean number of ACEs and among the modifiable variables, education, household income, smoking, and chronic illness had the strongest effects on the association between the number of ACEs and employment in adulthood. Public health strategies may target these variables to help improve employment among those with ACEs. In addition, based on research linking poor SES among parents with their children having increased risk of ACEs, improving adult employment among those with ACEs can help reduce ACEs in future generations.

## Data Availability

Publicly available datasets were analyzed in this study. This data can be found at: BRFSS data are available on the CDC, https://www.cdc.gov/brfss/data_documentation/index.htm.
